# MAIT Cells Detect and Efficiently Lyse Bacterially-Infected Epithelial Cells

**DOI:** 10.1371/journal.ppat.1003681

**Published:** 2013-10-10

**Authors:** Lionel Le Bourhis, Mathilde Dusseaux, Armelle Bohineust, Stéphanie Bessoles, Emmanuel Martin, Virginie Premel, Maxime Coré, David Sleurs, Nacer-Eddine Serriari, Emmanuel Treiner, Claire Hivroz, Philippe Sansonetti, Marie-Lise Gougeon, Claire Soudais, Olivier Lantz

**Affiliations:** 1 Institut curie, Inserm U932, Paris, France; 2 Avenir Group Inserm UMR925, Amiens, France; 3 Institut Pasteur, Unité de Pathogénie Microbienne Moléculaire, U786, Paris, France; 4 Institut Pasteur, Unité Immunité Antivirale, Biothérapies et Vaccins, Paris, France; 5 Center of Clinical Investigations CICBT507 IGR/Curie, Paris, France; 6 Equipe labellisée de la ligue de lutte contre le cancer, Institut Curie, Paris, France; National Institute of Allergy and Infectious Diseases, National Institutes of Health, United States of America

## Abstract

Mucosal associated invariant T cells (MAIT) are innate T lymphocytes that detect a large variety of bacteria and yeasts. This recognition depends on the detection of microbial compounds presented by the evolutionarily conserved major-histocompatibility-complex (MHC) class I molecule, MR1. Here we show that MAIT cells display cytotoxic activity towards MR1 overexpressing non-hematopoietic cells cocultured with bacteria. The NK receptor, CD161, highly expressed by MAIT cells, modulated the cytokine but not the cytotoxic response triggered by bacteria infected cells. MAIT cells are also activated by and kill epithelial cells expressing endogenous levels of MRI after infection with the invasive bacteria *Shigella flexneri.* In contrast, MAIT cells were not activated by epithelial cells infected by *Salmonella enterica Typhimurium*. Finally, MAIT cells are activated in human volunteers receiving an attenuated strain of *Shigella dysenteriae*-1 tested as a potential vaccine. Thus, in humans, MAIT cells are the most abundant T cell subset able to detect and kill bacteria infected cells.

## Author Summary

Human Mucosa-Associated Invariant T cells (MAIT) detect microbe-derived compounds presented by the MHC-like molecule, MR1. These foreign antigens are produced by a wide variety of microbes, including commensal and pathogenic bacteria or yeasts. MAIT cells expend shortly after birth and constitute the major antibacterial T cell subset described and, hence, could play important roles in infectious diseases. Here we show that MAIT cells recognize epithelial cells infected by the intestinal pathogen *Shigella flexneri* in a process requiring endogenous MR1, while the closely related bacterium *Salmonella Tyhpimurium* is not. Upon recognition, infected epithelial cells are efficiently lysed by MAIT cells. We also show that the triggering of CD161, a natural killer receptor highly expressed by MAIT cells, can modulate the cytokine but not the cytotoxic function of these cells. Finally, we provide evidence that MAIT cells are activated during the course of an experimental enteric infection in humans. Our study provides important insight on the antibacterial function of MAIT cells and their interaction with pathogenic bacterial species.

## Introduction

Detection of bacterial infections occurs through detection of microbial compounds by the innate immune receptors [1], [2]. As the infection progresses, the adaptive immune system respond to compounds produced by these pathogens in a process that requires priming of naïve cells and subsequent proliferation and differentiation. Innate like T cells bridge these two systems by providing immediate effectors functions in response to the infection [3], [4]. Indeed, in contrast to conventional T cells that express a very diverse T cell receptor (TCR) repertoire and are restricted by polymorphic MHC molecules, innate like T cells display semi-invariant TCRs and are restricted by non-polymorphic MHC-Ib molecules. In humans, they represent large oligoclonal expansions with immediate effector properties. Within the innate-like T cells, Mucosa-Associated Invariant T (MAIT) cells are restricted by the evolutionarily conserved MHC related molecule, MR1 [5], [6]. In humans, MAIT cells are abundant in peripheral blood and liver, are uniformly memory and display a tissue-targeted phenotype [7], [8]. MAIT cells express transcription factors associated with specific effector activities such as RORγt and ZBTB16 [7], [8]. Accordingly, they express at their cell surface high levels of cytokine receptors for IL-18, IL-12 and IL-23 [8], [9]. MAIT cells functions are probably related to their capacity to secrete TNF-α, IFN-γ, IL-17 as well as Granzyme B [8], [10], the latter suggesting cytotoxic capability. MAIT cells are identifiable by the high expression of CD161 and the detection of the Vα7.2 TCRα segment [8], [9]. CD161 is a C-type lectin-like membrane receptor and is also known as NKR-P1A. The ligand of CD161 is the lectin-like transcript 1 (LLT1) [11], which is detected on activated B cells and TLR-activated pDC and cDCs [12]. Whether CD161 triggering has activatory or inhibitory effects is still not clear [12], [13] and its impact on MAIT cell functions is not known.

MAIT cells detect highly conserved compounds derived from bacteria and yeasts, which confer them with a wide specificity to microbes [10], [14], [15]. These compounds have been recently identified as derivatives of riboflavin precursors synthesized by most microbes [15]. The MR1 molecule presenting these coumpounds is ubiquitously expressed [16], hence many cell types could have the capacity to activate MAIT cells including non-phagocytic epithelial cells. Bacterial pathogens induce their own uptake in these cells, providing a way to enter the host organisms through epithelial surfaces [17]. For example, *Shigella flexneri* (Sf), *Salmonella enterica* serovar *Typhimurium* (ST) and *Listeria monocytogenes* (Lm) are intestinal pathogens, which inject effector proteins that induce internalization of the bacteria through a phagocytic-like mechanism [17]. While ST mainly remains in a vacuole that does not fuse with the lysosomal compartment, Sf and Lm escape to the cytoplasm and then to neighboring cells [17]. As the MAIT specific ligand belongs to the riboflavin metabolic pathway [15], which is present in *Enterobacteriacea* [15], commensal species such as *Escherichia coli* as well as pathogens like *Shigella* and *Salmonella* can provide the MAIT specific ligand. However, *Listeria* species do not have this metabolic pathway, providing an explanation for their lack of MAIT stimulatory potential [10], [14], [15]. Although, these pathogens are known to induce T cell responses when presented by hematopoietic cells, the question remains whether MAIT cells sense their presence in epithelial cells.

In this study, we show that MAIT cells can kill epithelial cells presenting a bacterial ligand on MR1. Interestingly, the NK receptor molecule, CD161 modulates the cytokine response after triggering but does not abrogate the cytotoxic activity of MAIT cells. MAIT cells recognize and effectively lyse epithelial cells infected by Sf, in a process requiring only endogenous levels of MR1. In contrast, MAIT cells do not sense ST-infected epithelial cells. Moreover, MAIT cells become activated and decreased in numbers in the blood of human volunteers who had been orally vaccinated with an attenuated strain of *Shigella dysetenriae*-1 as a candidate vaccine. Therefore, MAIT cells are abundant innate like T cells that can detect and clear infected cells early in bacterial infections.

## Results

### MAIT cells are cytotoxic

**Figure 1:**
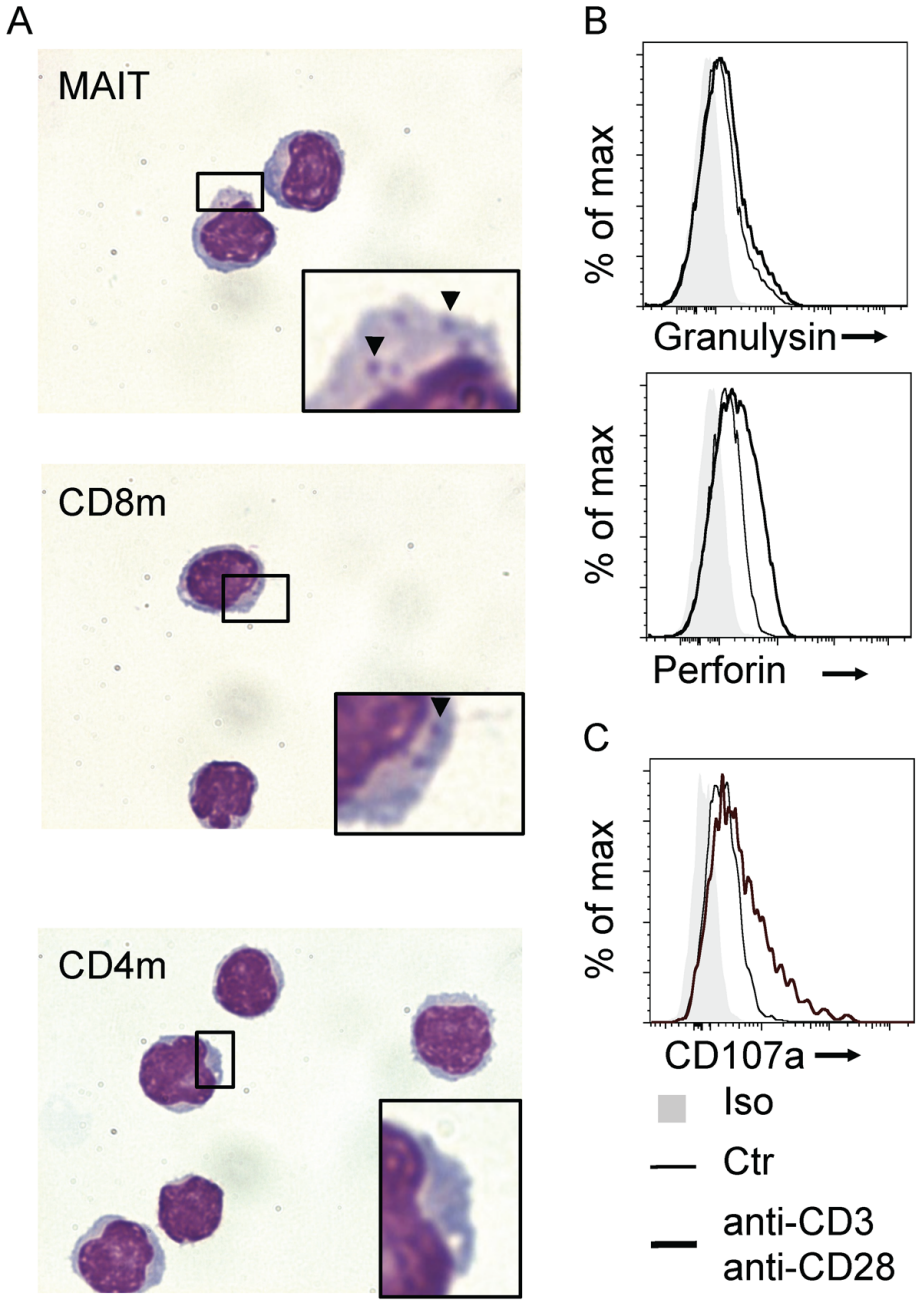
Human MAIT cells have a cytotoxic potential. (A) May-Grunwald Giemsa coloration of sorted MAIT (Vα7.2^+^CD161^hi^), CD8 and CD4 memory (CD45RO^+^) T cells from peripheral blood. Arrows indicates basophilic granules present in the cytoplasm of CD8 memory T cells and MAIT cells. (B) Intracellular staining of Granulysin and Perforin and (C)surface staining of CD107a (LAMP1) in purified MAIT cells in resting conditions (thin black lines) or after stimulation (anti-CD3-CD28 antibody, heavy black lines) compared to isotype control (grey). Results are representative of two independent experiments.

The expression of CD8 coreceptors (αβ heterodimer or αα homodimer) by MAIT cells [9] suggests the expression of a differentiation program with cytotoxic potential. This is confirmed by the secretion of granzyme A and B after TCR triggering [8]. Accordingly, May-Grunwald-Giemsa-staining of FACS sorted MAIT cells revealed basophilic granules that are very similar to those found in non-MAIT memory CD8^+^ T cells whereas these granules were absent from memory CD4^+^ T cells (Figure 1A). Additionally, intra-cytoplasmic staining of MAIT cells for cytotoxic molecules, granulysin and perforin was positive at steady state in comparison with isotype control (Figure 1B). Upon stimulation with anti-CD3 -CD28 beads, staining for perforin increased whereas granulysin levels were barely modified (Figure 1B). MAIT degranulated upon stimulation as surface staining for CD107a (LAMP1) increased. Taken together, these observations show that MAIT cells express the necessary molecules to be cytotoxic.

**Figure 2:**
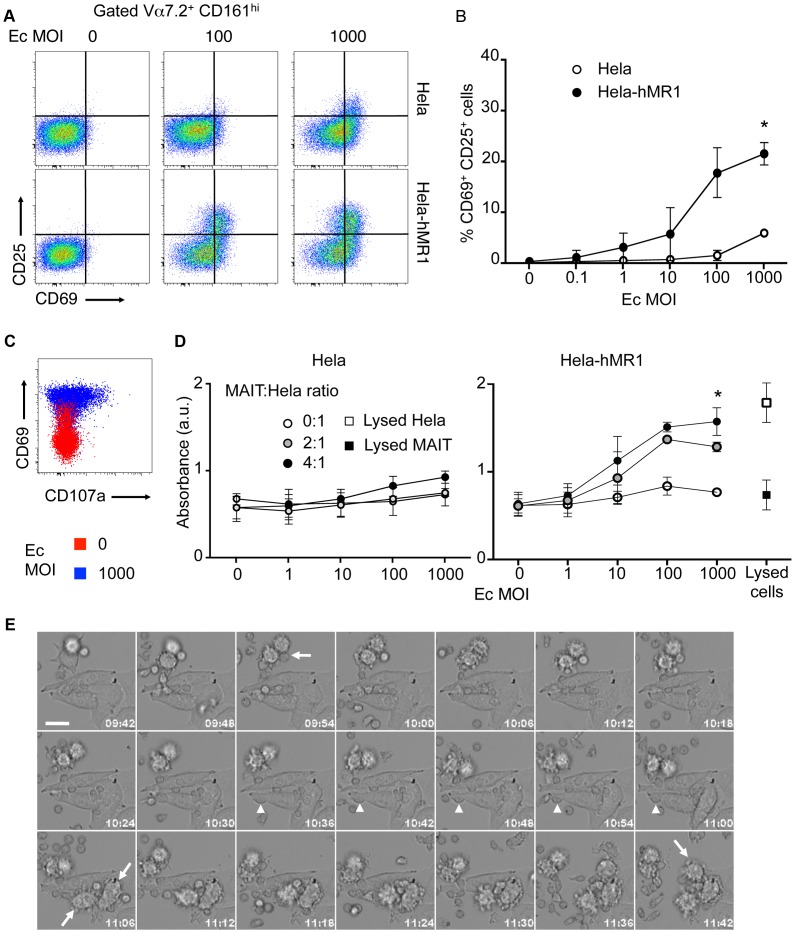
Human MAIT cells are cytotoxic. (A–B) Activation of purified MAIT (Vα7.2^+^CD161^hi^) cells cultured with Hela cells or Hela cells overexpressing the human MR1 protein (Hela-hMR1), in presence of increasing multiplicity of infections (MOI) of *Escherichia coli* (Ec). (A) FACS analysis of CD69 and CD25 upregulation. (B) Data are mean and SEM of three independent experiments as in A. * indicates statistical significance for the 2 parameters (MOI dose-response curve and Hela vs Hela-MR1) by 2 way ANOVA. (C) Degranulation assessed by CD107a (LAMP1) staining at the cell surface (red: uninfected control; blue: MOI of 1000). (D) Cytotoxicity of MAIT cells cultured with Hela or Hela-hMR1 cells as in A, with several effector∶target ratios (MAIT∶Hela), assessed by quantification of LDH release in the supernatants after overnight coculture. Maximum LDH release after chemical-induced lysis from Hela-hMR1 cells or MAIT cells is represented (white and black squares, respectively). Data are mean and SEM of two independent experiments. * indicates statistical significance for the 2 parameters (MOI dose-response curve and presence of MAIT or not) by 2 way ANOVA. (E) MAIT cells direct cytotoxicity was visualized by time-lapse analysis of Hela-hMR1 cells cultured in presence of Ec bacterial lysate and sorted MAIT (Vα7.2^+^CD161^hi^) cells. Arrows indicate cells entering apoptosis. Arrowheads indicate MAIT cells in prolonged interactions with a target cell. Time indicated on each frame (h∶m). Scale bar: 20 µm.

However, this cytotoxic potential has not been tested to date. We used epithelial cell line, overexpressing human MR1, as target cells (Hela-hMR1). Hela-hMR1 cells express at their cell surface a high level of MR1, which is normally more present in the endosomal compartment [18]. We cultured these cells and the parental cell lines (Hela) with increasing multiplicity of infection (MOI) of the non-invasive bacteria, *Escherichia coli* (Ec). After washing, FACS-sorted MAIT cells (Vα7.2^+^ CD161^hi^ T cells) were seeded on these presenting cells. After overnight incubation, MAIT cells activation was assessed by upregulation of CD69 and CD25 at their surface by FACS analysis. Ec cultured with Hela-hMR1 activated MAIT cells in a dose dependent manner (Figure 2A–B). This activation was significantly lower with endogenous level of MR1 in parental Hela cells and was only visible at high MOI (Figure 2A–B). These results were repeated in another cell type, HT-1080 with similar results (Figure S1). In the conditions where MAIT cells are highly activated, CD107a presence at the cell surface was detected (Figure 2C), indicative of degranulation processes in these cells (Figure 2C). To test the specific killing of target cells by MAIT cells in presence of bacteria, we quantified the release of lactate dehydrogenase (LDH), a cytoplasmic protein, in the culture supernatant. We observed a MOI-dependent increase of LDH in the culture supernatant of Hela-hMR1 cells in the presence of MAIT cells and bacteria (Figure 2D). The LDH release also depended on the effector/target ratio as more MAIT cells per Hela-hMR1 cells increased LDH amounts (Figure 2D). The induction of LDH release in parental Hela cells was limited and the cell death induced by Ec in the absence of MAIT cells was negligible (Figure 2D). To visualize the killing events, we performed time-lapse microscopy. Hela and Hela-hMR1 cells were cultured with FACS sorted MAIT cells, in the presence of Ec lysates or not, and frame-by-frame analysis was performed overnight. We observed that Ec lysates induced killing events observed when the lymphoid smaller cells entered in contact with bigger epithelial Hela cells. These killing events were numerous in conditions with Hela-hMR1 and the lysate, but very rare with Hela cells and totally absent in conditions without of the bacterial lysate (Figure 2E, Movie S1, S2, S3, S4). Taken together, these results clearly demonstrate that MAIT cells are cytotoxic, killing target cells in a MR1 and bacterial ligand dependent manner.

### CD161 modulates cytokine secretion but spares cytotoxicity of MAIT cells

**Figure 3:**
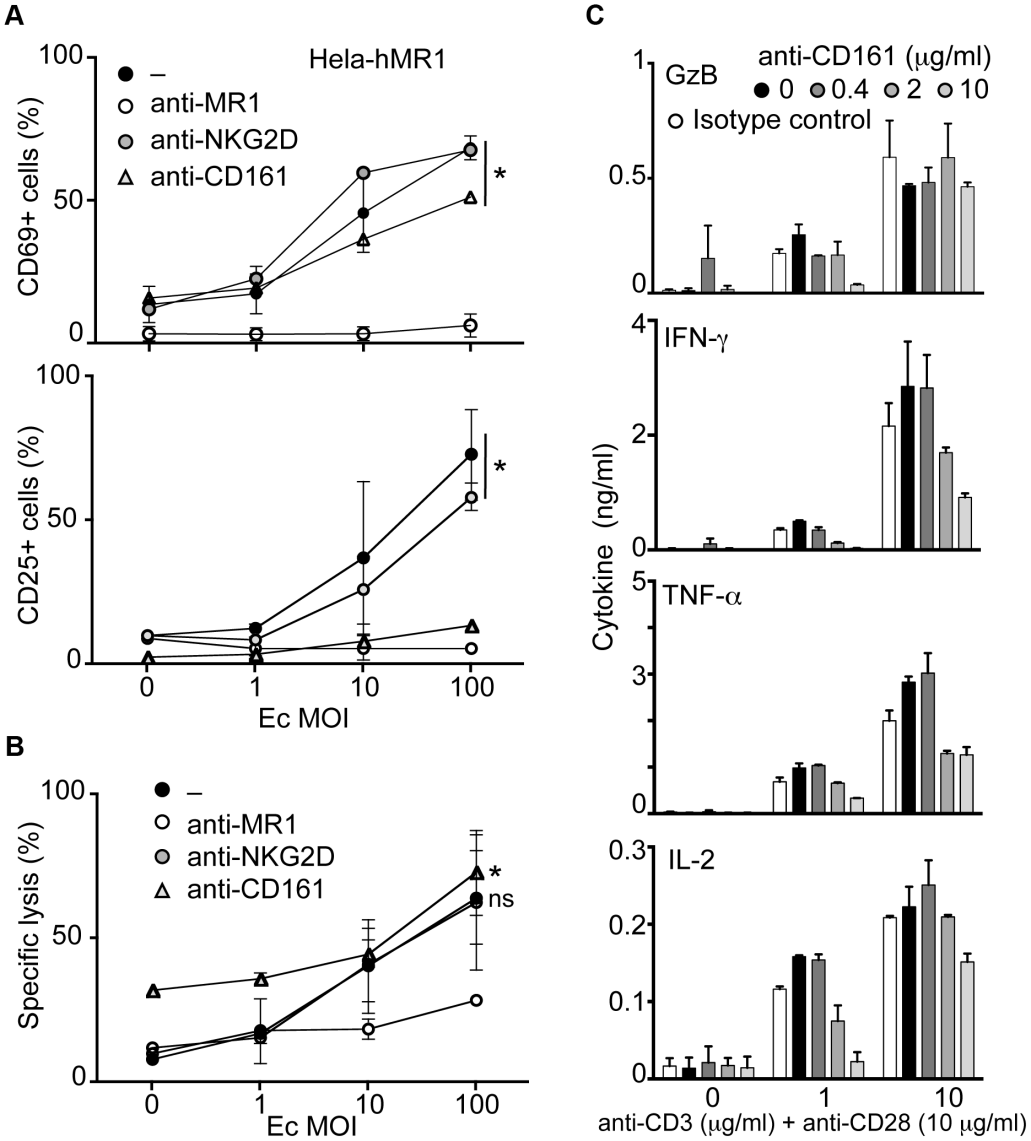
CD161 triggering inhibits the cytokine secretion but spares the cytotoxic response of MAIT cells. (A–B) MACS sorted MAIT cells were activated by Ec-infected Hela-hMR1 in presence of control, anti-MR1 (10 µg/ml), anti-NKG2D (10 µg/ml) or anti-CD161 antibody (10 µg/ml). (A) Upregulation of CD69 and CD25 was assessed by FACS and (B) the cytotoxic effect of MAIT cells by LDH release. Data are mean and SEM of two independent experiments. * indicates statistical significance for the MOI dose-response curve, while “ns” indicates the absence of effect of anti-NKG2D or anti-CD161 treatment by 2 way ANOVA. (C) MAIT cells were FACS sorted using anti-Vα7.2 and anti-IL18Rα before being stimulated with increasing doses of plastic-coated anti-CD3 (as indicated) and anti-CD28 (10 µg/ml) monoclonal antibodies in the presence of increasing doses of anti-CD161 antibody. Cytokines were quantified after 24 h stimulation. Data are mean and SEM of two independent experiments.

MAIT cells express high levels of CD161 (NKRP1A) and NKG2D [8], [9]. Ligation of CD161 induces inhibitory signals on NK cells [19] whereas it can be co-stimulatory on T cells [12]. Triggering NKG2D induces effector functions in T cells [20]. Hence, we tested the modulation of MAIT cell responses towards bacteria infected epithelial cells in the presence of specific antibodies against these receptors. In presence of anti-NKG2D antibody, the MAIT cell response to Hela-hMR1 cells cultured with increasing amounts of Ec was unaffected, with upregulation of CD69 and CD25 comparable to control. The presence of anti-MR1 antibody abrogated this response. Surprisingly, in the presence of anti-CD161 antibody, the upregulation of CD25 was reduced. However, levels of CD69 were similar to the control conditions, indicating that MAIT cell response was not fully inhibited (Figure 3A).

In the same conditions, as estimated by LDH release, anti-NKG2D antibody did not change the cytotoxic capacities of MAIT cells. Interestingly, anti-CD161 antibody did not inhibit the MAIT dependent killing of Hela-hMR1 infected cells either. However, anti-MR1 antibody clearly reduced LDH release from target cells (Figure 3B). These results indicate that modulation of CD25 upregulation did not impair the capacity of MAIT cells to be cytotoxic. Taken together, these observations suggest that the ligation of CD161 modulates the MAIT cell response.

We hypothesized that CD161 cross-linking might modify the cytokine response of MAIT cells induced by TCR triggering. To test this hypothesis in an analytical setting, we FACS sorted MAIT cells gating on the Vα7.2^+^, CD4 negative and IL-18Rα^hi^ cells as an alternative sorting strategy to avoid CD161 triggering prior to the experiments [8]. We then stimulated MAIT cells with increasing doses of plastic-coated anti-CD3 and anti-CD28 in the presence of increasing amounts of soluble anti-CD161 antibody. The production of cytokines was assessed after 24 hours in the culture supernatant. The presence of anti-CD161 antibody reduced the amounts of IFN-γ, TNF-α and IL-2 in a dose dependent manner (Figure 3C). Interestingly, Granzyme B secretion was not modified by CD161 triggering at high concentration of anti-CD3+CD28 antibodies (Figure 3C). As controls, no difference was observed with an irrelevant antibody (anti-NKG2D) but increase amounts of IFN-γ and TNF-α were detected in presence of soluble IL-12 (data not shown). Taken together, these data suggest that CD161 cross-linking modulates the cytokine but not the cytotoxic response of MAIT cells.

### MAIT cells do not sense intracellular *Salmonella enterica Typhimurium* in epithelial cells

We then tested the potential relevance of the cytotoxic ability of MAIT cells by studying an infection with the invasive bacteria *Salmonella enterica Typhimurium* (ST). Previous works have used ST to study the MAIT cell response to infected cells, however these studies relied on cell lines over-expressing hMR1 [15], [21]. As we have shown previously [10] and above, MR1 over-expressing-cells can present the MAIT cell specific ligand from non-invasive bacteria such as Ec when used at high MOI. Hence, we tested if ST detection by MAIT cells required epithelial cell invasion.

**Figure 4:**
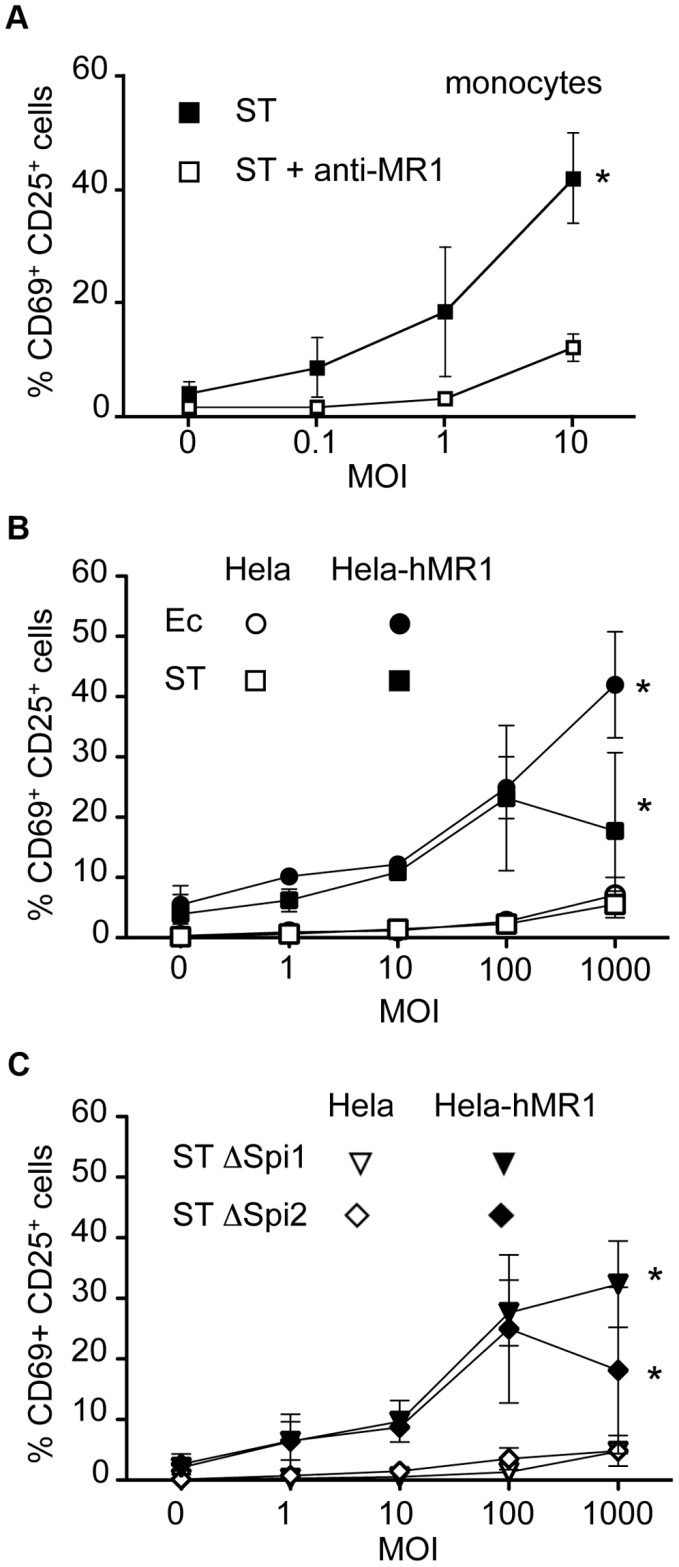
Epithelial cells infected by invasive *Salmonella Typhimurium* do not activate MAIT cells. (A) MAIT cells are activated by autologous monocytes cultured in the presence of PFA-fixed bacteria (*Salmonella enterica* serovar *Typhimurium* (ST)) as assessed by CD69 and CD25 upregulation by Vα7.2^+^CD161^hi^ cells. Activation was blocked by adding to the culture an anti-MR1 antibody (10 µg/ml). Data are mean and SEM of two independent experiments. * indicates statistical significance for the MOI dose-response curve by 2 way ANOVA. (B) MAIT cells recognize Hela-hMR1 but not Hela cells infected by Ec or ST. Hela and Hela-hMR1 cells were infected by Ec or ST for 30 min before gentamicin treatment to kill extracellular bacteria. Then MACS sorted Vα7.2^+^ cells were cocultured with infected cells overnight. MAIT cell activation was assessed by CD69 and CD25 upregulation of by Vα7.2^+^CD161^hi^ cells. Data are mean and SEM of three independent experiments. (C) ST ΔSpi1 and ST ΔSpi2 strains were used to infect Hela and Hela-hMR1 cells as in (B). Then MACS sorted Vα7.2^+^ cells were added for overnight culture. MAIT cells activation was assessed by upregulation of CD69 and CD25. Data are mean and SEM of three independent experiments.

We first confirmed the presence of the MAIT ligand in this Salmonella strain by feeding PFA-fixed ST to monocytes. By fixing the bacteria, monocytes were less sensitive to the cell death induced by the pathogenic bacteria, but the capacity of the bacteria to activate MAIT cells was preserved [10]. Autologous MAIT cells cultured in the presence of these ST-infected monocytes overnight displayed CD69- CD25-upregulation, indicative of a strong activation (Figure 4A). This activation was blocked by the addition of the anti-MR1 antibody (Figure 4A), confirming the specificity of the reaction. These results confirm the presence of the MAIT specific ligand in ST.

We then performed a gentamicin treatment assay with the invasive strain of ST on Hela cells over-expressing or not hMR1. After 30 minutes of infection in absence of serum and antibiotics to ensure bacterial fitness and allow invasion, cells were washed extensively and placed in medium supplemented with serum and gentamicin, which kill extracellular bacteria. Magnetic sorted Vα7.2 positive T cells were then added for overnight culture. Surprisingly, ST-infected Hela cells failed to induce a strong response from MAIT cells, only inducing upregulation of CD69 and CD25 at high MOI, to levels comparable to Ec (Figure 4B). As previously shown [15], [21], Hela-hMR1 cells in the presence of ST activated MAIT cells but at similar levels than Ec (Figure 4B). The lower activation observed at high MOI with ST is probably related to a cytotoxic effect of ST. We checked by cell lysate plating on LB agar plates and by immunofluorescence that ST was properly invasive (data not shown and Figure S2). These results suggest that ST-infected epithelial cells are not more potent MAIT cell activator than Ec-infected cells.

*Salmonella* entry is mediated by the injection of effector proteins through a molecular syringe, all of which are encoded by a region of the bacterial chromosome, *Salmonella* pathogeny island 1, Spi1 [22]. We compared ST deficient for the Spi1 locus, which do not enter epithelial cells, to the wild type strain for their capacity to activate MAIT cells. We did not find any difference in the upregulation of activation markers after overnight culture of MAIT cells with wild-type- or ΔSpi1-infected Hela or Hela-hMR1 cells (Figure 4B–C). On the other hand some virulence factors critical for ST pathogeny are encoded by a second locus, Spi2. This mutant is not virulent in vivo and is defective for its intracellular trafficking in epithelial cells [23], hence these bacteria could allow for MAIT cell dependent sensing. However, no difference was observed when comparing the activation of MAIT cells by wild-type- or ΔSpi2-infected Hela or Hela-hMR1 (Figure 4B–C). Taken together, these results show that intracellular *Salmonella* does not induce stronger activation of MAIT cells than non-invasive bacteria.

### MAIT cells detect epithelial cells infected by *Shigella flexneri*

*Salmonella* invasion of epithelial cells shares many features with *Shigella* mechanisms of entry, however the following steps of infection differ [17]. While Sf escape to the cytoplasm, ST reside in a vacuole preventing its fusion with the lysosomal compartment. We therefore tested the capacity of MAIT cells to detect Sf-infected cells.

**Figure 5:**
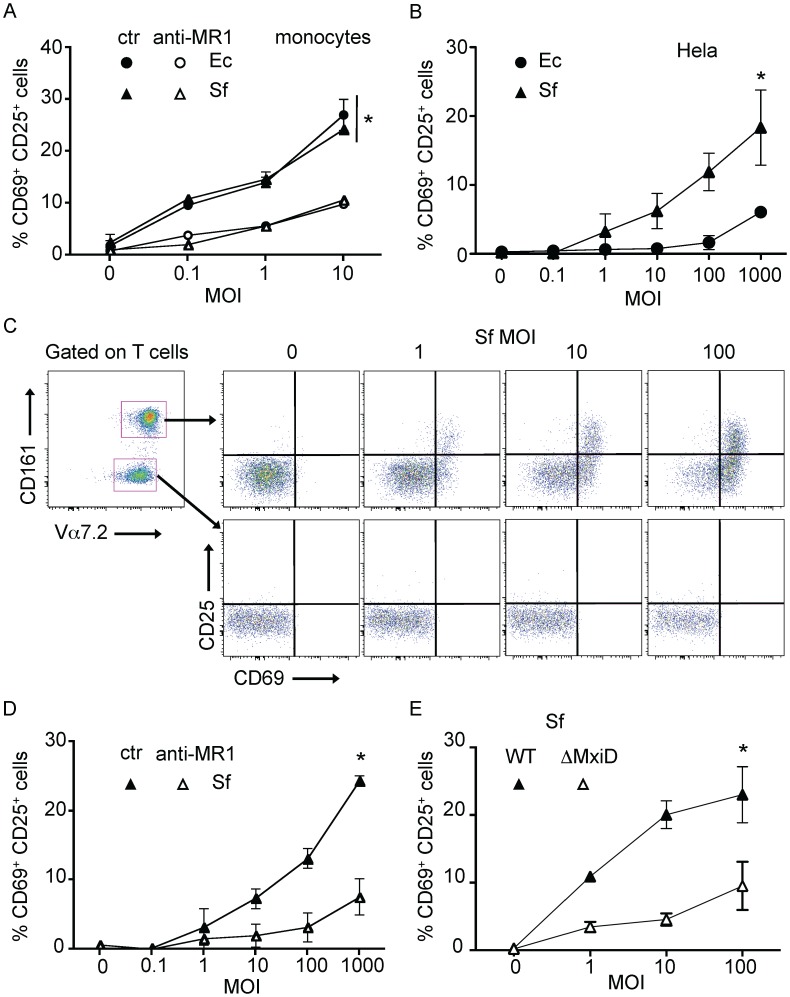
Epithelial cells infected by invasive *Shigella flexneri* activate MAIT cells. (A) MAIT cells are activated by autologous monocytes cultured in the presence of PFA-fixed bacteria (Ec, or *Shigella flexneri* (Sf)). MACS sorted Vα7.2^+^ cells where cultured overnight in presence of monocytes with increasing MOI of fixed bacteria, activation was assessed by CD69 and CD25 upregulation of by MAIT cells (Vα7.2^+^CD161^hi^). Activation was blocked by adding an anti-MR1 antibody (10 µg/ml) into the culture. Data are mean and SEM of two independent experiments. * indicates statistical significance for the MOI dose-response curve by 2 way ANOVA. (B) MAIT cells are activated by Hela cells infected with invasive Sf but not the non-invasive Ec strain. Hela cells were put in presence of increasing MOI of live Ec or Sf, before adding gentamicin to kill extracellular bacteria. MACS sorted Vα7.2^+^ cells were added overnight and activation of MAIT cells (Vα7.2^+^CD161^hi^) was assessed by CD69 and CD25 upregulation. Data are mean and SEM of three independent experiments. (C) MAIT cells activation by Hela cells infected as in (B) with Sf was specific. Only Vα7.2^+^CD161^hi^ (MAIT) cells and not Vα7.2^+^CD161^neg^ conventional T cells were activated in the same conditions. Representative of more than three experiments. (D) MAIT activation by Sf-infected Hela cells as in (B) is MR1-dependent as addition of the anti–MR1 (10 µg/ml) antibody prevents upregulation of CD69 and CD25. Data are mean and SEM of three independent experiments. (E) MAIT activation requires invasion of Hela cells by Sf, as the none-invasive ΔMxiD mutant is unable to induce upregulation of CD69 and CD25. Data are mean and SEM of two independent experiments.

We first checked the presence of the MAIT specific ligand in Sf. To do so, we used monocytes cultured in presence of PFA-fixed Ec or Sf. After overnight co-culture with autologous MAIT cells, we observed increased CD69 and CD25 expression by these T cells (Figure 5A). The Ec- and Sf-dependent activations were MR1 dependent as the addition of an anti-MR1 antibody abrogated the upregulation of CD69 and CD25 (Figure 5A). These results clearly indicate that Sf expresses the MAIT specific ligand.

We then studied the response of MAIT cell towards epithelial cell expressing physiological levels of MR1 in the context of Sf invasion. We infected non-transfected Hela cells with increasing doses of Sf or Ec, washed and co-cultured with Vα7.2 positive sorted cells. MAIT cell activation was assessed by upregulation of CD69 and CD25 by Vα7.2^+^CD161^hi^ cells. Sf-infected Hela cells activated MAIT cells in a MOI dependent manner (Figure 5B–C) and significantly more than Ec-infected cells (Figure 5B). This activation was specific to MAIT cells, as the Vα7.2^+^CD161^neg^ T cells cultured in the same conditions were not activated (Figure 5C). This activation was blocked by the addition of the anti–MR1 antibody (Figure 5D), indicating that endogenous level of MR1 present in the parental Hela cell line is sufficient for MAIT cell activation.

Finally, we compared wild type Sf with an uninvasive mutant, ΔMxiD. This virulence protein controls the secretion of the effector proteins necessary for Sf entry into epithelial cells [24]. The ΔMxiD Sf mutant induced significantly less MAIT cell response as compared with wild type Sf (Figure 5E). The response induced was comparable to the activation observed with Ec (Figure 5B). These results indicate that bacterial invasiveness is required for the strong MAIT cells activation induced by Sf. Taken together, these results show that MAIT cells detect *Shigella*-infected epithelial cells in a manner requiring cell invasion and presentation of the specific ligand on endogenous MR1.

**Figure 6:**
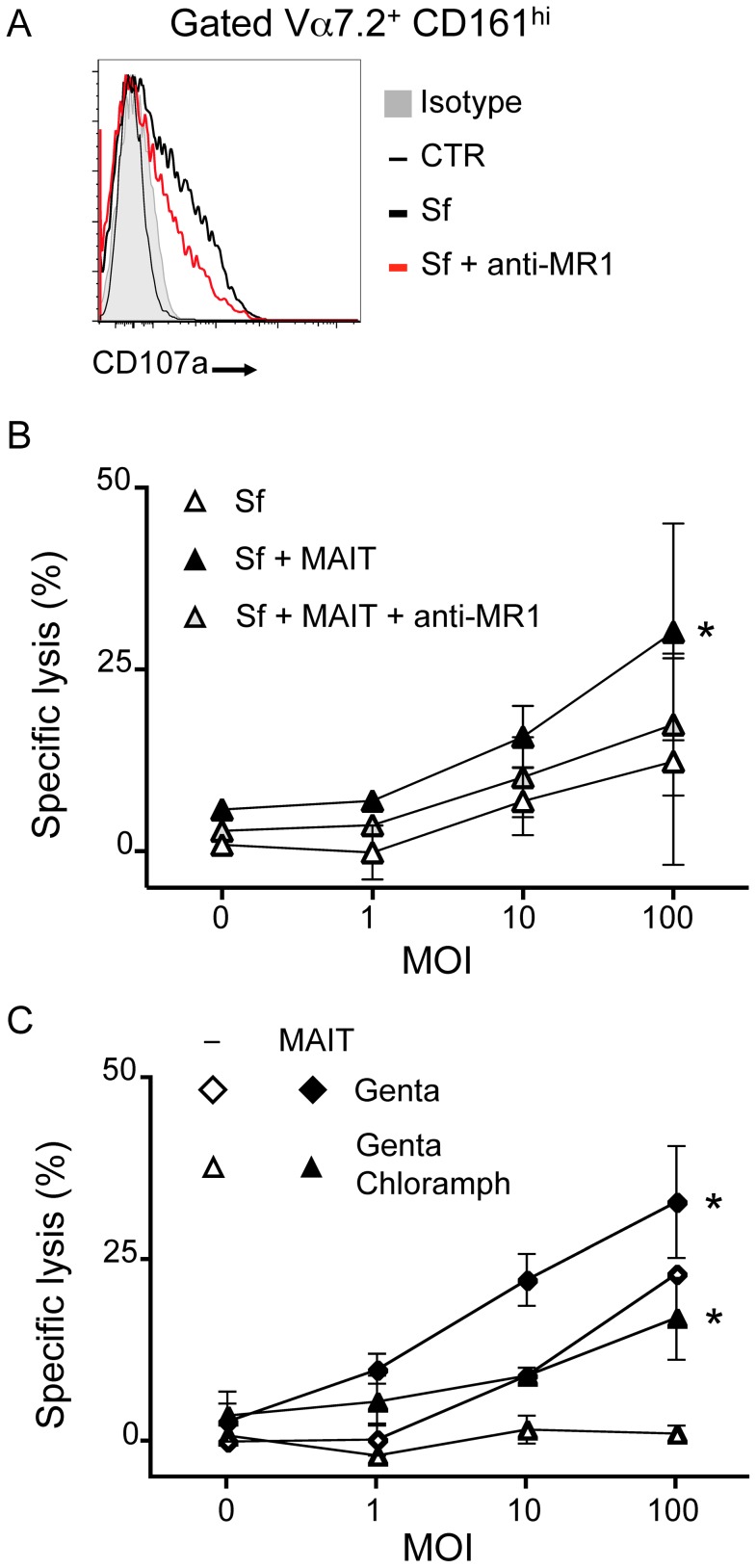
MAIT cells lyse *Shigella flexneri*-infected epithelial cells. (A) MAIT cells cultured with Sf-infected Hela cells (as in 5B) degranulate as observed by presence of CD107a at their cell surface (grey: isotype control; thin black line: uninfected; bold black line: Sf MOI 100; red line: Sf MOI 100 with anti-MR1). Representative of two independent experiments. (B) MAIT cells are cytotoxic to Sf-infected Hela cells. Sf-infected Hela cells were cocultured with MACS sorted Vα7.2^+^ cells and LDH release assessed after overnight culture. LDH release was increased in presence of MAIT cells as compared with Sf-infected Hela alone. This increase is blocked by addition of the anti–MR1 antibody (10 µg/ml). Data are mean and SEM of two independent experiments. * indicates statistical significance for the MOI dose-response curve by 2 way ANOVA. (C) MAIT cell-dependent cytotoxicity is additional to the one induced by the pathogenic Sf. Hela cells infected by Sf were put in medium supplemented with gentamicin (Genta) as in 5B or with both gentamicin and chloramphenicol (Genta Chloramph) before coculture with MACS sorted Vα7.2^+^ cells. LDH release from target cells was assessed after overnight culture. Data are mean and SEM of two independent experiments.

The capacity of MAIT cells to lyse Sf-infected epithelial cells was then tested in the same conditions. MAIT cells stimulated by Sf-infected Hela cells degranulated upon activation as the CD107a marker was increased at the cell surface as compared with the uninfected condition (Figure 6A). The quantification of LDH release in the supernatant of overnight-infected cells revealed, as expected, that Sf infection induced cell death in a MOI dependent manner as compared to uninfected cells (Figure 6B). However, infected Hela cells cultured in the presence of MAIT cells showed increased LDH release compared with Hela cells alone, suggesting that, indeed, MAIT cells could lyse Sf-infected cells (Figure 6B). This increased LDH release was abrogated by the addition of the anti-MR1 antibody (Figure 6B), further supporting the hypothesis of a MAIT cell-dependent killing. To determine the contribution of the MAIT-induced cell death compared to the Sf-triggered cytotoxicity, we added to the classical gentamicin treatment, chloramphenicol, a cell-permeant antibiotic, which kill intracellular bacteria. Sf-dependent cell death was reduced in the gentamicin plus chloramphenicol treatment condition as compared with gentamicin only. The level of LDH release was similar to the uninfected control even with high Sf MOI (Figure 6C), indicating that bacterial viability is necessary for Sf-induced cell death. When MAIT cells were added in the same conditions, higher LDH activity was found in the supernatants of the infected cells in both types of antibiotic treatment as compared with Hela cells without T cells (Figure 6C). In the two conditions (gentamicin and gentamicin plus chloramphenicol), MAIT cell activation was similar (data not shown). These results indicate that MAIT cells lyse Sf-infected epithelial cells in an endogenous MR1-dependent manner.

### MAIT cells are activated after oral administration of a vaccine *Shigella* strain

We then studied the relevance of enteric invasive bacterial pathogen detection by MAIT cells *in vivo*. To do so, we analyzed MAIT cell numbers and phenotype in the blood of volunteers that participated in a clinical trial testing the efficacy of an attenuated strain of *Shigella dysenteriae 1* (SD1), SC599, as an oral vaccine [25]. In this study, healthy volunteers received orally 10^5^ or 10^7^ live bacteria. SD1-LPS-specific antibody secreting cell (ASC) response (IgA, IgG, IgM) as well as SD1-specific antibody response were measured in the blood and serum of these subjects and compared to individuals receiving a placebo. The vaccinated group could be divided into 2 subgroups: responders (R) who showed specific IgA response above the threshold of 20 ASC per 10^6^ PBMC and non-responders (NR) who did not [25].

**Figure 7:**
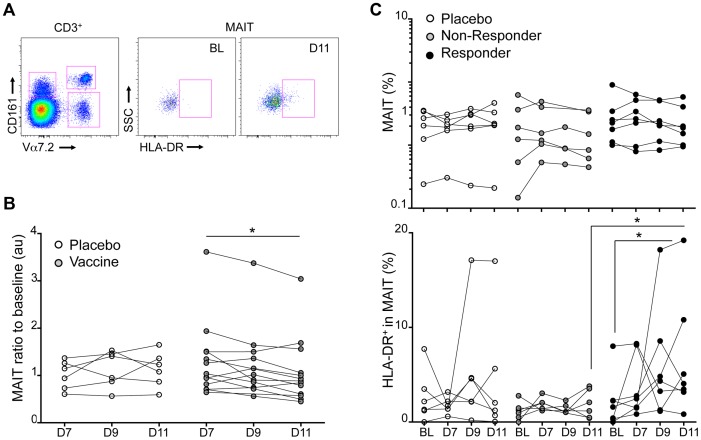
Experimental in vivo *Shigella flexneri*-infection results in MAIT cells activation. MAIT cell phenotype was analyzed in PBMCs from volunteers vaccinated with an attenuated strain of *Shigella dysenteriae 1* (SC599) and compared to subjects receiving a placebo. (A) Expression of the activation marker HLA-DR on MAIT cells at base line or D11. (B) MAIT cell percentages in the blood that have been normalized to the BL value of volunteers receiving a placebo (white symbols) or the vaccine. Each dot represents the value for a subject and the lines join the values obtained at the different time points. * indicates statistical significance between D7 and D11 by paired Wilcoxon t-test. (C) Upper panel: MAIT cell percentages of volunteers that have received the placebo or the vaccine segregated according to their B-cell response: Non Responders (grey symbols) and Responders (black symbols). Each dot represents the value for a subject and the lines join the values obtained at the different time points: at base line (BL), at day 7, 9 and 11 post vaccination. Lower panel: proportion of HLA-DR positive cells in the MAIT cell subset represented as above. * indicates statistical significance between BL and D11 by paired Wilcoxon t-test, or unpaired Mann-Whitney t-test between non-responder and responder at D11.

We measured the number of MAIT cells in the PBMCs from these volunteers at day 7, 9 and 11 (D7, D9 and D11) after vaccine administration as compared to baseline levels (BL) (Figure 7A). A significant reduction of the percentage of MAIT cells normalized to BL was observed at D11 in subjects receiving the bacteria as compared with the placebo group (Figure 7B). No differences were detected in other T cell subsets such as CD161^+^ T cells or Vα7.2^+^CD161^lo^ cells (Figure S3). These results suggest that circulating MAIT cell numbers are specifically modified after an oral experimental infection with *Shigella*.

When MAIT cells were analyzed separately in the R and NR groups, only a trend towards an increased MAIT number at BL was detected in the responding subjects (Figure 7C). However, in the responder group the MAIT cells were more activated at day D11 as shown by the significant upregulation of the activation marker HLA-DR in comparison with the BL or the non-responder group (Figure 7C). This activation state was not observed in the non-responder group (Figure 7C). Although one subject in the placebo group displayed MAIT cells with increased HLA-DR expression at D11 the difference was not significant for the group as a whole (Figure 7C). No significant expression of HLA-DR was detected in the CD161^+^ or Vα7.2^+^CD161^lo^ T cell subsets (Supplementary Figure S3). Taken together, these results strongly suggest that MAIT cells are specifically activated during the course of an enteric bacterial infection with a *Shigella dysenteriae* strain in human.

## Discussion

MAIT cells are microbial reactive CD8 T cells that produce effector molecules such as TNF-α, IFN-γ and IL-17 [8], [10]. However, their cytotoxic capacity has not been assessed to date. Our study demonstrates that MAIT cells can lyse MR1 expressing epithelial cells in the presence of bacteria or bacterial ligand. We show that the NK receptor CD161, which is highly expressed by MAIT cells, modulates the cytokine but not the cytotoxic response of this T cell subset. Furthermore, epithelial cells infected with the invasive bacteria *Shigella flexneri*, but not *Salmonella enterica Typhimurium*, are excellent targets for MAIT cell dependent cytotoxicity in a process depending on physiological levels of MR1. Finally, we show that MAIT cells are activated by the oral administration of *Shigella dysenteriae* in human volunteers that mounted a B-cell response towards the vaccine strain.

The cytotoxic capacity of MAIT cells, a large T cell population with a wide microbial reactivity, could have major impacts on many infectious diseases caused by pathogens expressing MAIT specific ligands [10], [15]. The lysis of infected cells could participate to the immune control of bacterial infections by limiting the spreading of the bacteria into the organisms. MAIT cells also secrete cytokines such as IFN- γ and TNF- α that could also be involved in this antibacterial response. This secretion was reduced in the presence of anti-CD161 antibody. The effect of CD161 triggering on NK or T cell effector activities is still controversial [19], [26]. A recent report suggests that CD161 has stimulatory function on IL-17 producing CD4 T cells [27]. However, an inhibitory effect was previously shown on NK cells and CD8 T cells [19], [27], [28]. Our data confirm this latter finding using a homogenous T cell population. This modulation of the cytokine response contrasting with an unaltered cytotoxic response could be an important mechanism to control MAIT cell response. We propose a model in which MAIT cell detect and lyse infected epithelial cells, preventing early propagation of the bacterial infection without inducing a strong inflammatory response. However if the bacterial infection goes further, phagocytic cells would pick up bacteria or infected cell remnants. In this case, activation of MAIT cells by these professional presenting cells producing IL-12 and IL-23 would induce the secretion of cytokines such as IFN-γ and IL-17, which increases the adaptive and innate immune response to the infection. The known CD161 ligand, LLT1, is expressed by activated antigen presenting cells. The expression of LLT1 by epithelial cells both in inflammatory and steady-state conditions remains to be elucidated and could have a significant impact on MAIT stimulation.

*Salmonella* block the fusion of the bacteria containing vacuole with the endosomal and lysosmal compartments. By mediating this inhibition, Salmonella could block the mechanisms that are necessary for efficient loading of the ligand into the MR1 groove. Interestingly, it was recently shown that *Salmonella* activate MAIT cells when cultured with non-phagocytic cells [15], [21], however in these studies, the presenting cells overexpress MR1 and the necessity for bacterial entry was not assessed. Additionally, no differences were observed between *Salmonella* and other non-invasive bacteria such as *Escherichia coli*, *Pseudomonas aeroginosa* or *Klebsiella pneumoniae* [21]. This discrepancy with our study could be explained by the overexpression of MR1. Our results show that the active entry into epithelial cells of *Salmonella* does not enhance MAIT activation. We can speculate that some *Salmonella* virulence factors are responsible for the absence of MR1 loading.

As the immune system and pathogens co-evolve, one has to always consider that the pathogen could use the immune response to its benefit. Considering the intracellular life-cycle of *Shigella*, we can hypothesize that the escape of the bacteria to the cytoplasm leads to an efficient loading of the MAIT ligand on MR1. The cellular and molecular mechanisms by which the MAIT specific ligand is loaded on MR1 need to be determined. However the recent determination of the nature of this compound and the use of intracellular bacterial pathogens should provide the tools to study these processes.

The results we report herein *in vivo* in human volunteers show that MAIT cells are activated after ingestion of the attenuated strain of *Shigella dysenteriae 1*, SC599 [25]. This activation was observed in the individuals that mounted a detectable B-cell response against the bacteria and not in the non-responders. The original study of this clinical trial [25] applied two doses of bacteria with no change in the antibody response, suggesting that the lack of immunogenicity is not due to a lack of antigen availability. However, shedding of the vaccine strain was detectable in only 20 to 30% of the feces of volunteers, suggesting that these results might be explained by the absence of sufficient uptake of the bacteria in the intestine. It remains to be assessed whether differences in MAIT cell response to the bacteria are implicated in the antibody response observed in this study [25].

In summary, our study shows that MAIT cells efficiently kill bacterially infected non-phagocytic cells. Modulation of the cytokine response of these bacteria specific T cells could be achieved by CD161 triggering. Finally, we show that MAIT cells participate to the immune response against an enteric infection in humans.

## Materials and Methods

### Human samples

Blood samples were obtained from healthy donors from the blood bank (Etablissement Français du sang, site de Crozatier) in accordance with institutional regulations.

PBMCs were obtained using a standard Ficoll gradient according to the manufacturer protocol (GE healthcare). Monocytes were isolated by adherence on plastic culture plates. MAIT cells were isolated by MACS sort using the biotinylated anti-Vα7.2 (3C10) antibody and anti-biotin magnetic beads (Miltenyi) according to the manufacturer specifications. In some experiments, the positive fraction was subsequently, stained with anti-CD3-A700 (HIT3a, BioLegend), anti-CD161-APC (191B8, Miltenyi Biotec), and streptavidin phycoerythrin (PE)-Cy7 (BD Biosciences PharMingen). MAIT cells were fluorescence-activated cell sorter sorted on a BD Aria II.

### Shigella vaccine study design and subject recruitment

This randomized, double-blind, placebo-controlled Phase 2 trial was conducted in healthy volunteers recruited into two vaccine trial centres, the “CIC de Vaccinologie Cochin-Pasteur” in Paris, France, and the St George's Vaccine Institute in London, United Kingdom in 2006. The protocol was approved by Local Ethics Committees and competent authorities, and a written informed consent was obtained from all volunteers. This study is registered at ClinicalTrials.gov with the identifier NCT00210288. Fasting volunteers ingested 120 ml of 2% sodium bicarbonate buffer, followed 5 min later by 30 ml 2% (w/v) bicarbonate solution containing the assigned vaccine dose or no vaccine (placebo). SC599 vaccine was an attenuated *Shigella dysenteriae* strain, described elsewhere [29]. Quantification by Elispot of circulating IgA-ASC (antibody secreting cells) was performed on days 0, 7, 9 and 11 after vaccination and responders to the vaccine were defined as exhibiting ≥20 LPS-specific IgA ASC/10^6^ PBMC. Complete details of the clinical trial has been published [25]. Cryopreserved PBMC from subjects included either in the placebo group, or in the group vaccinated with 10^7^ CFU were tested for peripheral MAIT cells frequency, as detailed below.

### Microbes, infection, activation and cytotoxicity

*Escherichia coli*, Dh5α ATCC strain was used as uninvasive bacteria. *Shigella flexneri* strain M90T and MxiD mutants were used throughout the study and was provided by Pr. Sansonnetti (Institut Pasteur, France). *Salmonella enterica Typhimurium* SL1344, ΔSpi1 and ΔSpi2 mutants were provided by Dr. Tedin (University of Berlin, Germany). Bacteria were cultured overnight in Luria broth at 37°C, then diluted 1/100 for a 3 hour subculture with shaking for *Shigella*, without for *Salmonella*, washed in PBS and diluted according to needs. Where indicated, bacteria were fixed in 1% PFA for 5 min and then washed extensively before use.

For *in vitro* infection, cells were washed and put in DMEM without supplement. Dilutions of bacteria in DMEM were added, spun down and left at 37°C for 30 minutes. Then cells were washed 3 times in complete medium and put back at 37°C for 2 hours in complete medium supplemented with 100 µg/mL Gentamicin (and 10 µg/mL Chloramphenicol where indicated). At this point, purified T cells were added for an overnight co-culture. Then cells were harvested and stained for FACS analysis.

The bacterial lysate was prepared by growing Dh5α to saturation then pellet the bacteria and resuspension to induce the release of the MAIT ligand (C. Soudais et al., in preparation).

Supernatants of cell culture were harvested and cytotoxic activity was assessed by quantification of the lactate dehydrogenase (LDH) activity. A commercial kit containing a colorimetric substrate was used according to the manufacturer specifications (Promega).

### Flow cytometry

Flow cytometry was performed with directly conjugated antibodies according to standard techniques with analysis on a FACS Aria and LSRII flow cytometers (Becton Dickinson). DAPI and a 405 nm excitation were used to exclude dead cells. The following antibodies, from BD Pharmingen, eBiosciences or Biolegend, for human cell staining, were used: anti-CD45RO-FITC (UCHL1), anti-CD4-APC-Cy7 (RP4-T4), anti-CD3ε-Alexa 700 (UCHT1), anti-CD8β-PE Texas Red (2ST8.5H7), anti-CD161-APC, -PE or -FITC (DX12), anti-CD69-APC or -PECy5 (CH/4) and the antibody anti-Vα7.2-biot, -PE or APC (3C10) has been described elsewhere.

For quantification of cytokines, CBA (BD biosciences) technology was used according to the manufacturer specifications.

### Time-lapse analysis

Hela cells, over-expressing MR1 or not, were cultured in presence of bacterial lysate, with enriched MAIT on 35 mm dishes (Fluorodish) placed into a chamber on the videomicroscope at 37°C in a 5% CO2 atmosphere. Time-lapse images were acquired on a Nikon Ti microscope equipped with a CCD camera (Roper Scientific), and a piezoelectric motor (LVDT, Physik Instrument), by using a dry 20× objective 1.40NA. Acquisition was done with the Metamorph software (MDS AZ). Stacks of z-planes (step 1 µm) were acquired every 3 min. Movies show the projection of the z stacks.

### Statistical analyses

All quantitative data were analyzed on Prism software using two-way ANOVA, testing the significance of the presence of bacteria (MOI) and the different conditions (Hela vs Hela-MR1; Sf vs Ec; or Ctr vs anti-MR1); * represents p<0.05 for both parameters. In figure 7, paired and unpaired nonparametric t-test was applied; * represents p<0.05.

## Supporting Information

Figure S1 HT1080 cells or HT1080 overexpressing hMR1 (HT1080-hMR1) cells were cultured in the presence of increase multiplicity of infection (MOI) of Escherichia coli (Ec). After washing and addition of an anti-MR1 or anti-CD161 antibody, MAIT cells were added for overnight culture. (A) Their activation was visualized by CD69 CD25 upregulation. (B) The specific killing of target cells was determined by lactate dehydrogenase (LDH) release.
(PDF)

Figure S2 Immunofluorescence analysis of bacteria uptake in Hela cells after Gentamicin treatment assay. (A) Hela cells infected with Shigella (green) were left alone (left) or cocultured with MAIT cells (CD3: blue) (right) and counter stained for actin (red). (B) Shigella (left) or Salmonella (right) were stained (green) after infection and incubation of the Hela cells with MAIT cells (Vα7.2: red). Counter-staining with DAPI (blue). Similar experiments were conducted with E. coli and no intracellular bacteria were detected.
(PDF)

Figure S3 PBMCs from volunteers receiving a Placebo (white symbols) or a vaccine strain of Shigella dysenteriae subdivided in Non-Responders (grey symbols) and Responders (black symbols) were analyzed for T cell numbers and HLA-DR expression. The two populations (Vα7.2^+^CD161^−^ and Vα7.2^−^CD161^+^ cells) showed no differences in percentage or activation markers comparing base line (BL) and days after vaccine ingestion (D7, D9 and D11) or between Placebo, Non-Responders and Responders.
(PDF)

Movie S1 MAIT cells were seeded on Hela cells and imaged every 3 min for 15 hours.
(MOV)

Movie S2 MAIT cells were seeded on hMR1 overexpressing Hela cells in the presence of bacteria lysate and imaged every 3 min for 15 hours.
(MOV)

Movie S3 MAIT cells were seeded on hMR1 overexpressing of Hela cells and imaged every 3 min for 15 hours.
(MOV)

Movie S4 MAIT cells were seeded on hMR1 overexpressing Hela cells in the presence of bacteria lysate and imaged every 3 min for 15 hours. Rounding of the much bigger epithelial cells indicating death is frequent only when both hMR1 and the bacteria lysate are present.
(MOV)
