# Supplementary material for: MAIT Cells Detect and Efficiently Lyse Bacterially-Infected Epithelial Cells
Source: PLoS Pathog. 2013 Oct 10;9(10):e1003681. doi: 10.1371/journal.ppat.1003681 (PMC3795036; doi:10.1371/journal.ppat.1003681)
Supplement: Figure S1 — HT1080 cells or HT1080 overexpressing hMR1 (HT1080-hMR1) cells were cultured in the presence of increase multiplicity of infection (MOI) of Escherichia coli (Ec). After washing and addition of an anti-MR1 or anti-CD161 antibody, MAIT cells were added for overnight culture. (A) Their activation was visualized by CD69 CD25 upregulation. (B) The specific killing of target cells was determined by lactate dehydrogenase (LDH) release. (PDF) [file ppat.1003681.s001.pdf]

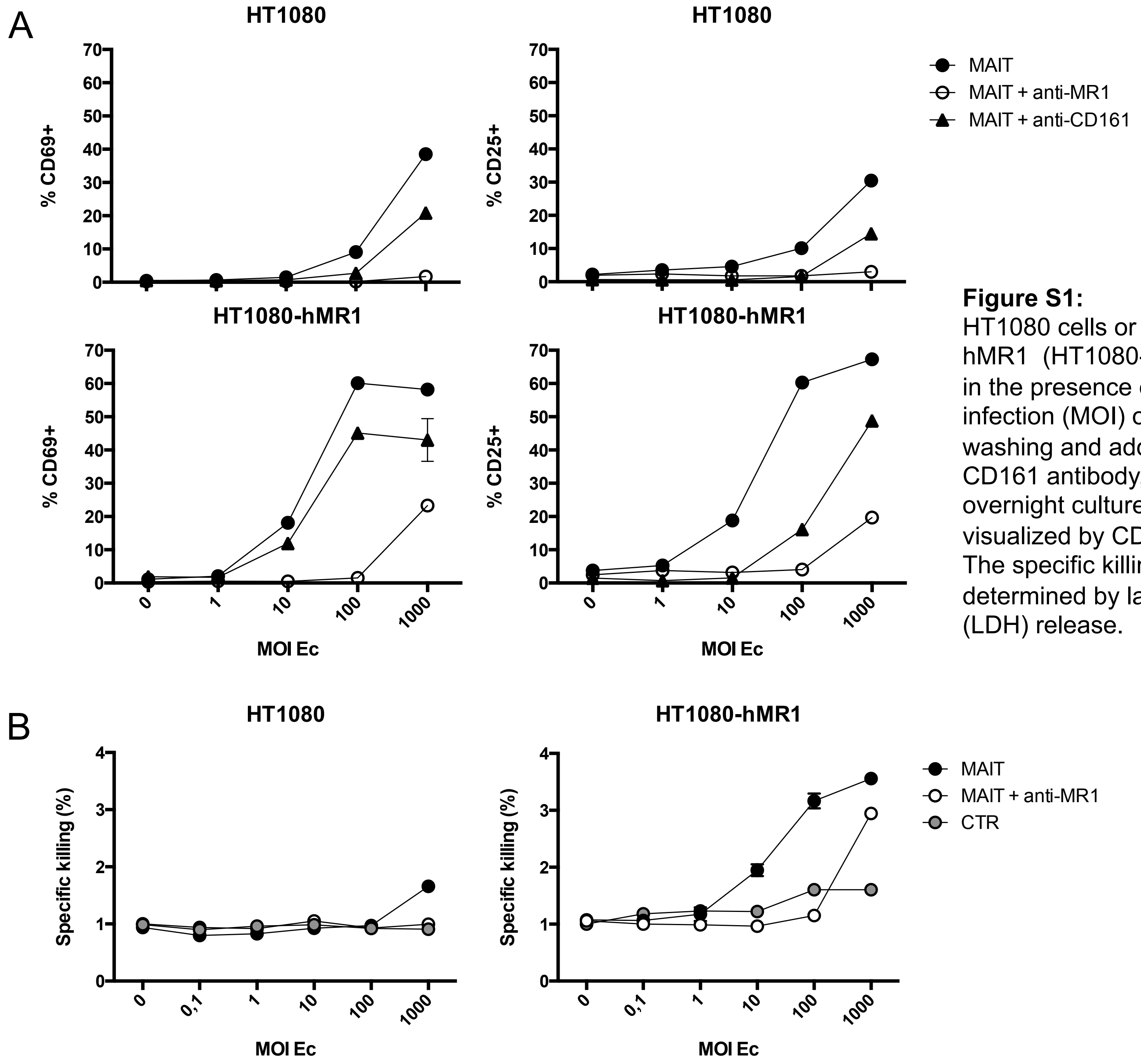

Figure S1:
HT1080 cells or HT1080 overexpressing hMR1 (HT1080-hMR1) cells were cultured in the presence of increase multiplicity of infection (MOI) of Escherichia coli (Ec). After washing and addition of an anti-MR1 or anti-CD161 antibody, MAIT cells were added for overnight culture. (A) Their activation was visualized by CD69 CD25 upregulation. (B) The specific killing of target cells was determined by lactate dehydrogenase (LDH) release.
