# Supplementary material for: MAIT Cells Detect and Efficiently Lyse Bacterially-Infected Epithelial Cells
Source: PLoS Pathog. 2013 Oct 10;9(10):e1003681. doi: 10.1371/journal.ppat.1003681 (PMC3795036; doi:10.1371/journal.ppat.1003681)
Supplement: Figure S2 — Immunofluorescence analysis of bacteria uptake in Hela cells after Gentamicin treatment assay. (A) Hela cells infected with Shigella (green) were left alone (left) or cocultured with MAIT cells (CD3: blue) (right) and counter stained for actin (red). (B) Shigella (left) or Salmonella (right) were stained (green) after infection and incubation of the Hela cells with MAIT cells (Vα7.2: red). Counter-staining with DAPI (blue). Similar experiments were conducted with E. coli and no intracellular bacteria were detected. (PDF) [file ppat.1003681.s002.pdf]

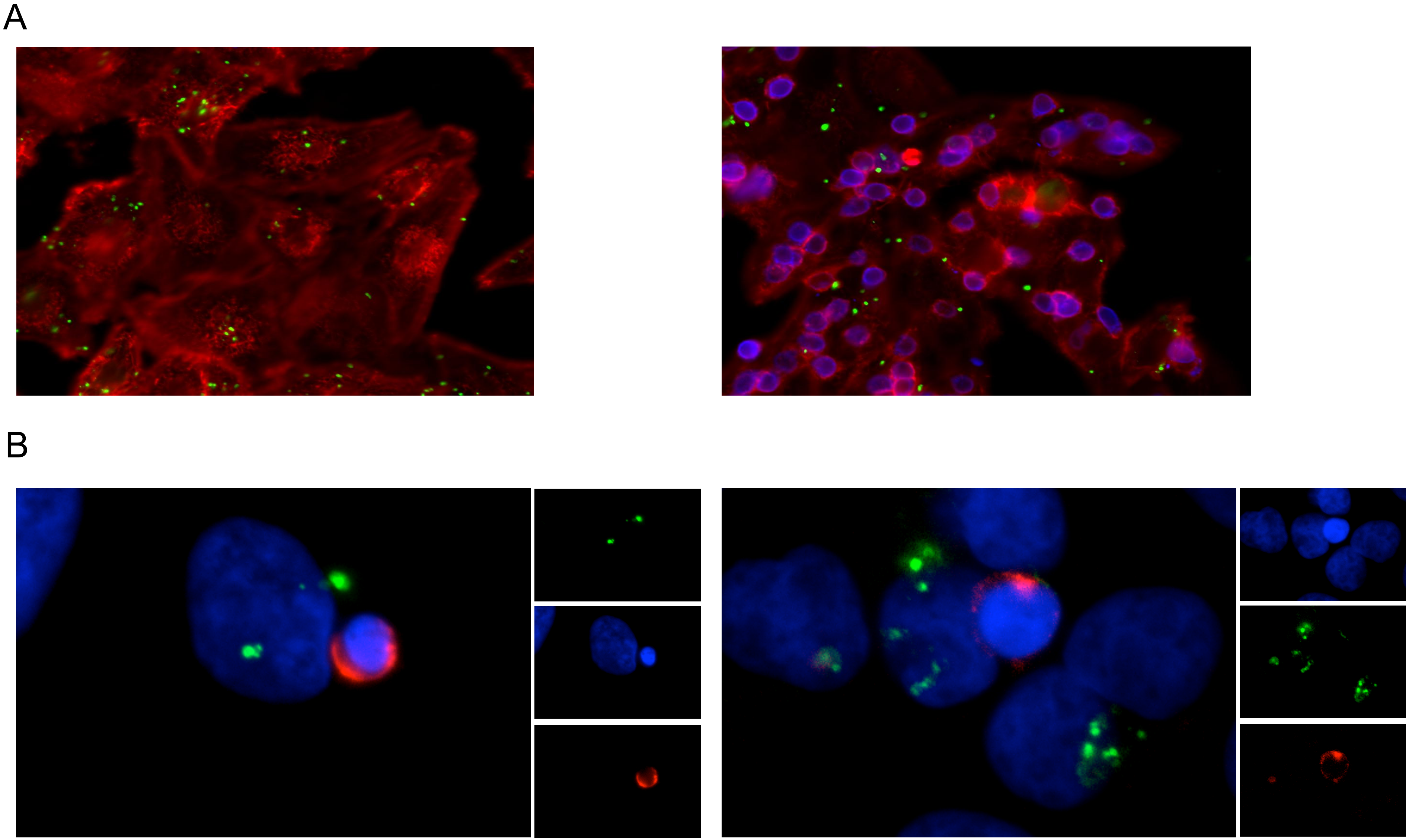

Figure S2:

Immunofluorescence analysis of bacteria uptake in Hela cells after Gentamicin treatment assay. (A) Hela cells infected with Shigella (green) were left alone (left) or cocultured with MAIT cells (CD3: blue) (right) and counter stained for actin (red). (B) Shigella (left) or Salmonella (right) were stained (green) after infection and incubation of the Hela cells with MAIT cells (Vα7.2: red). Counter staining with DAPI (blue). Similar experiments were conducted with E. coli and no intracellular bacteria were detected.
