# Supplementary material for: MAIT Cells Detect and Efficiently Lyse Bacterially-Infected Epithelial Cells
Source: PLoS Pathog. 2013 Oct 10;9(10):e1003681. doi: 10.1371/journal.ppat.1003681 (PMC3795036; doi:10.1371/journal.ppat.1003681)
Supplement: Figure S3 — PBMCs from volunteers receiving a Placebo (white symbols) or a vaccine strain of Shigella dysenteriae subdivided in Non-Responders (grey symbols) and Responders (black symbols) were analyzed for T cell numbers and HLA-DR expression. The two populations (Vα7.2+CD161− and Vα7.2−CD161+ cells) showed no differences in percentage or activation markers comparing base line (BL) and days after vaccine ingestion (D7, D9 and D11) or between Placebo, Non-Responders and Responders. (PDF) [file ppat.1003681.s003.pdf]

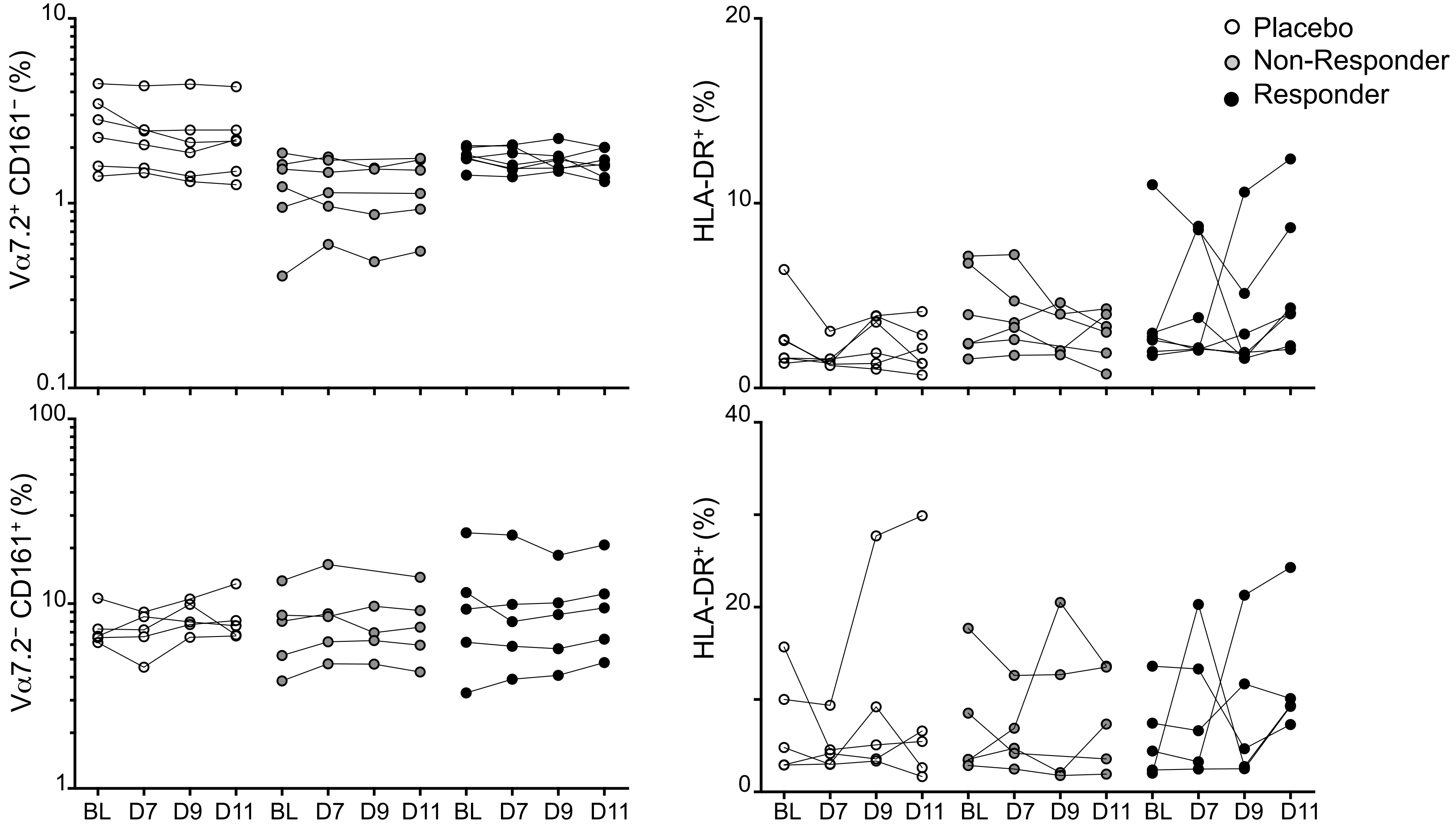

Figure S3:

PBMCs from volunteers receinving a Placebo (white symbols) or a vaccine strain of Shigella dysenteriae subdivided in Non-Responders (grey symbols) and Responders (black symbols) were analyzed for T cell numbers and HLA-DR expression. The two populations ($V\alpha7.2^{+}$ $CD161^{-}$ and $V\alpha7.2^{-}$ $CD161^{+}$ cells) showed no differences in percentage or activation markers comparing base line (BL) and days after vaccine ingestion (D7, D9 and D11) or between Placebo, Non-Responders and Responders.
